# Feature selection and transformation by machine learning reduce variable numbers and improve prediction for heart failure readmission or death

**DOI:** 10.1371/journal.pone.0218760

**Published:** 2019-06-26

**Authors:** Saqib E. Awan, Mohammed Bennamoun, Ferdous Sohel, Frank M. Sanfilippo, Benjamin J. Chow, Girish Dwivedi

**Affiliations:** 1 Department of Computer Science and Software Engineering, The University of Western Australia, Perth, Australia; 2 Discipline of Information Technology, Mathematics & Statistics, Murdoch University, Perth, Australia; 3 School of Population and Global Health, The University of Western Australia, Perth, Australia; 4 University of Ottawa Heart Institute, University of Ottawa, Ottawa, Canada; 5 Harry Perkins Institute of Medical Research and Fiona Stanley Hospital, The University of Western Australia, Perth, Australia; 6 Medical School, The University of Western Australia, Perth, Australia; University of Mississippi Medical Center, UNITED STATES

## Abstract

**Background:**

The prediction of readmission or death after a hospital discharge for heart failure (HF) remains a major challenge. Modern healthcare systems, electronic health records, and machine learning (ML) techniques allow us to mine data to select the most significant variables (allowing for reduction in the number of variables) without compromising the performance of models used for prediction of readmission and death. Moreover, ML methods based on transformation of variables may potentially further improve the performance.

**Objective:**

To use ML techniques to determine the most relevant and also transform variables for the prediction of 30-day readmission or death in HF patients.

**Methods:**

We identified all Western Australian patients aged 65 years and above admitted for HF between 2003–2008 in linked administrative data. We evaluated variables associated with HF readmission or death using standard statistical and ML based selection techniques. We also tested the new variables produced by transformation of the original variables. We developed multi-layer perceptron prediction models and compared their predictive performance using metrics such as Area Under the receiver operating characteristic Curve (AUC), sensitivity and specificity.

**Results:**

Following hospital discharge, the proportion of 30-day readmissions or death was 23.7% in our cohort of 10,757 HF patients. The prediction model developed by us using a smaller set of variables (*n* = 8) had comparable performance (AUC 0.62) to the traditional model (*n* = 47, AUC 0.62). Transformation of the original 47 variables further improved (p<0.001) the performance of the predictive model (AUC 0.66).

**Conclusions:**

A small set of variables selected using ML matched the performance of the model that used the full set of 47 variables for predicting 30-day readmission or death in HF patients. Model performance can be further significantly improved by transforming the original variables using ML methods.

## Introduction

Heart Failure (HF) is a prevalent cardiovascular disorder affecting more than 25 million people worldwide[1]. HF is also associated with a high rate of readmissions incurring significant economic costs, and driving healthcare policies to include financial penalties for hospitals that have high rates of readmissions for HF[2]. The adverse financial implications have served as a motivation to develop models that can accurately predict readmissions at the time of an index hospital discharge. These models have traditionally relied on patients’ historical data to predict the probability of a HF readmission[3].

The enormous amount of clinical and administrative data generated in the healthcare sector can be used to personalise care, improve the quality of treatment and reduce treatment costs. We and others have applied machine-learning (ML) techniques on administrative data previously to predict HF readmissions[4, 5]. These techniques use patient data to learn hidden patterns that significantly contribute to the outcomes. Predictive ability of the model depends on the variables selected. Since some variables may be redundant, a prediction model developed after selecting only the significant variables associated with the outcome, is expected to reduce the machine-training time and improve the predictive performance[6]. Moreover, such a prediction model will allow policy makers and healthcare workers to focus only on the minimum number of variables required to predict the outcome. Lastly, ML methods allow feature extraction, which is a process in which the original variables are transformed to create new variables that could provide superior performance in predicting outcomes compared to the model developed on the original variables [7].

The objective of the present study was to use ML techniques to determine the most clinically significant variables in administrative datasets that are associated with 30-day readmission or death in HF patients. We also evaluated if the variables transformed by feature extraction techniques could improve the performance of the developed ML models.

One of our previous studies investigated the ability of ML models to predict HF readmission and death [5]. That study showed that a large amount of medical data could be coupled with ML techniques to predict HF readmission and death with superior predictive performance than with standard regression methods. However, such prediction models are complex, and simpler models might be produced that can predict with similar accuracy. This can be done through feature selection algorithms. The objective of the present study was to use ML techniques to determine the most clinically significant variables in administrative datasets that are associated with 30-day readmission or death in HF patients and use them to develop simpler prediction models. We also evaluated if the variables transformed by feature extraction techniques could improve the performance of the developed ML models.

## Methods

### Ethics

This study was approved by the Human Research Ethics Committees of the Western Australian Department of Health (2014/11); the Australian Department of Health (XJ-16); and the University of Western Australia (RA/4/1/8065). We were granted a waiver of informed consent by each ethics committee. The linked administrative data received were fully anonymized, and the authors did not have access to any identifying data.

### Cohort and data sources

We used linked administrative data from the Hospital Morbidity Data Collection (HMDC) and death register which are two of the core datasets of the Western Australian Data Linkage System [8]. This is a dynamic linkage system based on probabilistic matching of records from multiple datasets with clerical review and quality control. The Western Australian Department of Health maintains these databases, with data supplied by all public and private hospitals in the state under obligatory reporting agreements. These were linked to data from the PBS for information on drug usage, and from the Medicare Benefits Scheme (MBS) for information on out-of-hospital services such as primary care visits. Details of the datasets and study cohort have been published previously [9].

We identified Western Australian patients aged 65 years and above who were hospitalised with a principal discharge diagnosis of HF (International Statistical Classification of Diseases and Related Health Problems 10^th^ Revision Australian Modification (ICD-10-AM) code I50) in 2003–2008 (index HF admission). We identified comorbidities and HF history by looking back 20 years from the date of the index HF admission, and outcomes of HF readmission or all-cause death by looking forward from the date of hospital discharge. We also identified the use of medications in the 6 months prior to the index HF admission from the linked PBS data, and out-of-hospital services from the linked MBS data.

### Collection of features (variables) for our cohort

We used the available 47 features related to patient demographics, admission characteristics, medical history, socio-economics, medication history, out-of-hospital healthcare services, emergency inpatient admission (S1 Table). We extracted these features from the linked datasets of HMDC, PBS, and MBS data.

### Feature (variable) selection

Feature selection is the process of choosing a subset of features, from a set of original features, based on a specific selection criteria [10]. The main advantages of feature selection are: 1) reduction in the computational time of the algorithm, 2) improvement in predictive performance, 3) identification of relevant features, 4) improved data quality, and 5) saving resources in subsequent phases of data collection.

#### Feature selection using the t-test

The outcome of interest was binary with two values: (i) 30-day HF readmission or death, and (ii) 30-day survival with no HF readmission. For a binary outcome, a significant difference in the values of a continuous input variable for each outcome value shows that the variable has the ability to distinguish between the two outcome values. We used the t-test to calculate the two-sided p value for the difference in means at the 5% level of significance. We retained those features with a two-sided p<0.05

#### Feature selection using Chi-Squared test

The chi-squared test determines whether there is a significant association between two categorical variables. We calculated the two-sided chi-squared p value to test the association between each categorical feature and the binary outcome at the 5% level of significance. We retained those features with a two-sided p<0.05.

#### Sequential forward selection

The sequential forward selection technique adds the next best feature to the feature-subset on a greedy basis. A greedy procedure always chooses the feature/variable that seems to give the best performance at that moment. A predetermined criterion such as mutual information or Pearson’s correlation coefficient is used to select the best feature. We used the mutual information to select the next best feature in this work. The selection process starts with an empty set and stops when a predefined threshold for the number of variables is reached [11]. In our work, the threshold was set to seven variables.

#### Sequential backward selection

The sequential backward selection technique removes one least important feature from the subset, based on a fixed criterion in every iteration. The selection process starts with a full set of features and stops when a predefined threshold for the number of variables is reached or further removal of features does not improve the performance [12]. We used mutual information as the selection criteria and set the selection threshold to seven variables. The performance was measured with the AUC.

#### The minimal redundancy maximal relevance feature selection

The mRMR technique ranks the variables based on their relevance to the target variable, and the redundancy between the variables themselves [13]. The relevance is characterized by the Mutual Information (MI), which is given as $I(v,c)=\iint p(v,c)log\frac{p(v,c)}{p(v)p(c)}dvdc$, where *v* is the input variable, *c* is the target variable, *p*(*v,c*) is the joint probabilistic density, and *p*(*v*) and *p*(*c*) denote the marginal probabilistic densities. The maximum relevance or dependence on the target variable is computed as, $D=\frac{1}{\left|S\right|}\sum_{v_{i}\epsilon S}I(v_{i},c)$. The variable with the largest D is termed the most relevant variable, which reflects a high dependency on the target variable. Variables with maximum relevance to the target can also be redundant. When two variables are redundant, the discriminatory power of the data does not change if we remove one of the redundant variables. The mRMR approach selects the mutually exclusive variables by minimizing the redundancy, given as $R=\frac{1}{{\left|S\right|}^{2}}\sum_{v_{i},v_{j}\epsilon S}I(v_{i},v_{j})$. Both relevance and redundancy are combined to generate the mRMR criterion, given as max(*D*−*R*). The input variable with the highest value of this criterion is selected at each iteration to produce a subset of the most important variables from the dataset.

### Feature (variable) extraction

This is the process of creating a new and a smaller set of variables, with the aim to capture the most useful information that is present in the original variables, to predict the outcome. The new variables are produced by applying a transformation to the original variables. The transformed variables represent projections of the original variables onto a new variable space, where the distinct outcome groups have a better separation compared to the original variable space.

#### Principal Component Analysis

Principal Component Analysis is a popular feature extraction method that creates a linear transformation of the input variables[14]. The new variables, called the principal components, are the projections of the original variables to a new variable space. The dimensions of the new variables can be reduced by sorting them based on their eigenvalues and selecting the variables with larger variance[15, 16]. A common choice is to select the first *m* components such that the variance of *m* components is 95% of the total variance that is present in the data[17]. For PCA, we first normalised the data to zero mean. Then we computed the covariance matrix, which measures the joint variability across the variables in a dataset. The covariance matrix contains covariance scores for every variable with every other variable including itself. The next step was to find the eigenvectors of the covariance matrix. Finally, the original data was multiplied with the eigenvectors to produce the transformed data.

### Machine learning model

We divided our cohort into three sets of data (70% for training, 15% for validation, and 15% for testing over unseen data) to build a generalized ML model for 30-day HF readmission or death. We tested the model performance using three measures: AUC, sensitivity and specificity. We used an MLP based model[18] to assess the performance of the feature selection and feature extraction techniques for HF readmission or death. We have previously shown that the MLP model performs best with these datasets. This model contained an input layer, one hidden layer of 50 nodes, and an output layer of one node. The hyper-parameters were chosen by a trial and test method. We chose a rectified linear unit as the non-linear transformation in the hidden layer and a sigmoid function for the output layer. The number of nodes (*n* = 50) in the hidden layer were empirically chosen to yield the best performance. We tested a wide range of hyper-parameters for the feature selection and feature extraction techniques, and present the results of the configuration that gives the best performance.

### Software packages

The programming codes used in this work were written in Python programming environment v3.6. The MLP model was implemented using the Keras Library v2.1.5 with Tensor Flow back end v1.8.0. We used the Scikit-learn library v0.19.1 to perform the PCA. The statistical comparison of AUCs was performed on SAS software v9.4.

## Results

Table 1 shows the patient characteristics of demographics, socio-economic indicators, medication history, deaths, medical history, and out-of-hospital services for the study cohort of 10,757. The proportion of 30-day HF readmission or death was 23.7% (*n* = 2546). The mean age was 82 years (Standard Deviation (SD) 7.6), and the average length of hospital stay during the index HF admission was 11.7 days (SD 26.7). Common comorbidities included diabetes (30%), hypertension (67%), atrial fibrillation (42%), chronic kidney disease (26%), ischaemic heart disease (55%), and chronic obstructive pulmonary disease (28%). About half of the patients (46%) had at least one emergency admission during the six months prior to the index HF admission. The mean Charlson comorbidity index was 4.3 (SD: 3.0).

**Table 1:** Characteristics of heart failure patients in the study cohort (n = 10,757).

Characteristic			
	Alive and not readmitted for HF within 30 days	Readmitted for HF or died within 30 days	Combined
Total number of HF patients (%)	8211 (76.3)	2546 (23.7)	10757 (100)
Age (years), mean (SD)	81.1 (7.6)	83.1 (7.6)	81.6 (7.6)
Males (%)	4028 (49.0)	1247 (49.0)	5275 (49.0)
Indigenous Status: Aboriginal and Torres Strait Islander (%)	141 (1.7)	35 (1.4)	176 (1.6)
History of heart failure	3642 (44.3)	1422 (55.8)	5064 (47.0)
Length of stay (days), mean (SD, median, IQR)	10.4 (15.9, 6.0, 9.0)	16.2 (46.8, 7.0, 14.0)	11.7 (26.7, 6.0, 10.0)
**Comorbidities (%)**			
Ischaemic heart disease	4506 (54.9)	1457 (57.2)	5963 (55.4)
Hypertension	5497 (66.9)	1751 (68.8)	7248 (67.3)
Atrial fibrillation	3398 (41.4)	1102 (43.3)	4500 (41.8)
Diabetes	2458 (29.9)	806 (31.6)	3264 (30.3)
Chronic obstructive pulmonary disease	2240 (27.3)	783 (30.7)	3023 (28.1)
Peripheral vascular disease	1547 (18.8)	549 (21.5)	2096 (19.4)
Stroke	1014 (12.3)	366 (14.4)	1380 (12.8)
Dementia	545 (6.6)	270 (10.6)	815 (7.5)
Depression	691 (8.4)	272 (10.7)	963 (8.9)
Cancer	2811 (34.2)	934 (36.7)	3745 (34.8)
Chronic kidney disease	2027 (24.7)	795 (31.2)	2822 (26.2)
Cardiogenic shock	68 (0.8)	30 (1.1)	98 (0.9)
Cardiomyopathy	344 (4.2)	115 (4.5)	459 (4.2)
**SEIFA (%)**			
5^th^ quintile (Least disadvantage)	552 (6.7)	182 (7.1)	734 (6.8)
4^th^ quintile	1398 (17.0)	439 (17.2)	1837 (17.0)
3^rd^ quintile	1450 (17.6)	474 (18.6)	1924 (17.9)
2^nd^ quintile	1810 (22.0)	555 (21.8)	2365 (22.0)
1^st^ quintile (Most disadvantage)	3001 (36.5)	896 (35.2)	3897 (36.2)
**ARIA residential location (%)**			
Major cities	4247 (51.7)	1334 (52.4)	5581 (51.9)
Inner regional	2514 (30.6)	692 (27.1)	3206 (29.8)
Outer regional	897 (10.9)	327 (12.8)	1224 (11.4)
Remote	330 (4.0)	118 (4.6)	448 (4.2)
Very Remote	223 (2.7)	75 (2.9)	298 (2.8)
**History of drugs in the last 6 months (%)**			
No supply of BB or RASI/ARB	2842 (34.6)	897 (35.2)	3739 (34.7)
1 or more supplies of RASI/ARB only	3335 (40.6)	1114 (43.8)	4449 (41.4)
1 or more supplies of BB only	669 (8.2)	189 (7.4)	858 (8.0)
1 or more supplies of both BB and RASI/ARB	1365 (16.6)	346 (13.6)	1711 (15.9)
**At least one visit to health professionals in the 6 months prior to index HF admission**			
GP	6929 (84.4)	2131 (83.7)	9060 (84.2)
Specialist	3980 (48.8)	1079 (42.4)	5059 (47.0)
Diagnostic	6556 (79.8)	2028 (79.6)	8584 (79.8)
Allied Health	1384 (16.8)	353 (13.9)	1737 (16.1)
At least one emergency inpatient admission in 6 months prior to index HF admission	3591 (43.7)	1303 (51.1)	4894 (45.5)
Charlson comorbidity score, mean (SD)	4.2 (3.0)	4.8 (3.1)	4.3 (3.0)
**At least 2 supplies of drugs from any ATC group in the 6 months prior to index HF admission**			
Alimentary tract and metabolism	4868 (59.3)	1660 (65.2)	6528 (60.7)
Blood and blood forming organs	3835 (46.7)	1183 (46.5)	5018 (46.6)
Cardiovascular system	7190 (87.6)	2251 (88.4)	9441 (87.8)
Dermatologicals	730 (8.9)	253 (9.9)	983 (9.1)
Genito urinary system and sex hormones	388 (4.7)	123 (4.8)	511 (4.8)
Systemic hormonal preparations, excl. Sex hormones and insulins	974 (11.9)	356 (14.0)	1330 (12.4)
Anti-infectives for systemic use	3101 (37.8)	1071 (42.1)	4172 (38.8)
Antineoplastic and immunomodulating agents	276 (3.4)	111 (4.3)	387 (3.6)
Musculo-skeletal system	2390 (29.1)	770 (30.2)	3160 (29.4)
Nervous system	4883 (59.5)	1674 (65.7)	6557 (61.0)
Antiparasitic products, insecticides and repellents	243 (2.9)	79 (3.1)	322 (3.0)
Respiratory system	1977 (24.0)	616 (24.2)	2593 (24.1)
Sensory organs	1950 (23.7)	656 (25.8)	2606 (24.2)
HF readmission within 30 days (emergency only)	1121 (10.4)		
Death (all-cause) within 30 days	1574 (14.6)		

ARB = Angiotensin Receptor Blockers; ARIA = Accessibility Remoteness Index of Australia; ATC [19] = Anatomical Therapeutic Chemical index; BB = Beta Blocker; GP = General Practitioner; HF = Heart Failure; RASI = renin angiotensin system inhibitor; SD = standard deviation; SEIFA = Socio-Economic Indexes for Areas.

Variables selected by the statistical and ML techniques for the prediction of 30-day HF readmission or death are listed in Tables 2 and 3, respectively.

**Table 2:** Variables selected by the statistical method (t-test and chi-squared test) for predicting 30-day HF readmission or death (sorted in ascending order of p-value).

Variables	p-value
**t-test**	
Age (years)	<0.0001
Length of stay (days)	<0.0001
Charlson comorbidity score	<0.0001
Time (days) since last HF discharge	0.0013
Socio-economic status	0.1654
Number of admissions in 6 months prior to index HF admission	0.3068
**Chi-squared test**	
History of HF	<0.0001
History of dementia	<0.0001
History of chronic kidney disease	<0.0001
Emergency inpatient admission in 6 months prior to index HF admission	<0.0001
Out-of-hospital visit to specialist	<0.0001
At least 2 supplies of drugs for alimentary tract and metabolism (ATC group A) in 6 months prior to index HF admission	<0.0001
At least 2 supplies of drugs for the nervous system (ATC group N) in 6 months prior to index HF admission	<0.0001
Index HF admission type (emergency/booked)	<0.0001

HF = Heart Failure; ATC [19] = Anatomical Therapeutic Chemical index.

**Table 3:** Variables selected by the wrapper-based machine learning techniques for predicting 30-day HF readmission or death.

Selection Technique	Selected variables
**Forward**	age, type of index HF admission, history of cancer and stroke, time (days) since last HF discharge, at least 2 supplies of systemic hormonal drugs in the last 6 months (ATC group H), at least 2 supplies of antineoplastic and immunomodulating agents in the last 6 months (ATC group L), use of beta blockers or RASI/ARB in the last 6 months.
**Backward**	type of index HF admission, out-of-hospital visit to allied health professional, at least 2 supplies of antineoplastic and immunomodulating agents in the last 6 months (ATC group L), at least 2 supplies of nervous system drugs in the last 6 months (ATC group N), an emergency inpatient admission in the last 6 months, history of dementia, HF and shock.
**mRMR**	age, type of index HF admission, out-of-hospital visit to allied health professional, length of hospital stay, at least 2 supplies of antineoplastic and immunomodulating agents in the last 6 months (ATC group L), history of chronic kidney disease, depression and HF.

ARB = angiotensin receptor blockers; ATC [19] = Anatomical Therapeutic Chemical index; HF = Heart Failure; mRMR = minimal Redundancy Maximum Relevance.

The most discriminatory continuous variables identified by the t-test in the statistical model (p<0.05) include age, length of hospital stay, time (days) since last HF discharge, and Charlson comorbidity index (Table 2). The chi-squared test identified the most prominent binary variables associated with the outcome. These included history of HF, dementia and chronic kidney disease, an emergency admission in the 6 months prior to the index HF admission, out-of-hospital visit to a specialist, use of alimentary tract and metabolism drugs and nervous system drugs in the 6 months prior to index HF admission, and the type of index HF admission (emergency or booked).

Variables selected by the forward and backward selection approaches in the ML model (Table 3) included age, type of index HF admission, time (days) since last HF discharge, history of HF, visits to allied health professionals, use of beta blockers or RASI/ARBs in the last 6 months, use of antineoplastic and immunomodulating drugs, use of systemic hormonal drugs, use of nervous system drugs, emergency inpatient admission in last 6 months and comorbidities of cancer, stroke, dementia, and shock. The variables which have maximum relevance to the outcome and minimum redundancy between the variables are: age, type of index HF admission, visit to allied health professional, length of hospital stay, at least two supplies of antineoplastic and immunomodulating drugs in the last 6 months, history of chronic kidney disease, depression, and HF.

### Machine-learning methods

**Fig 1:**
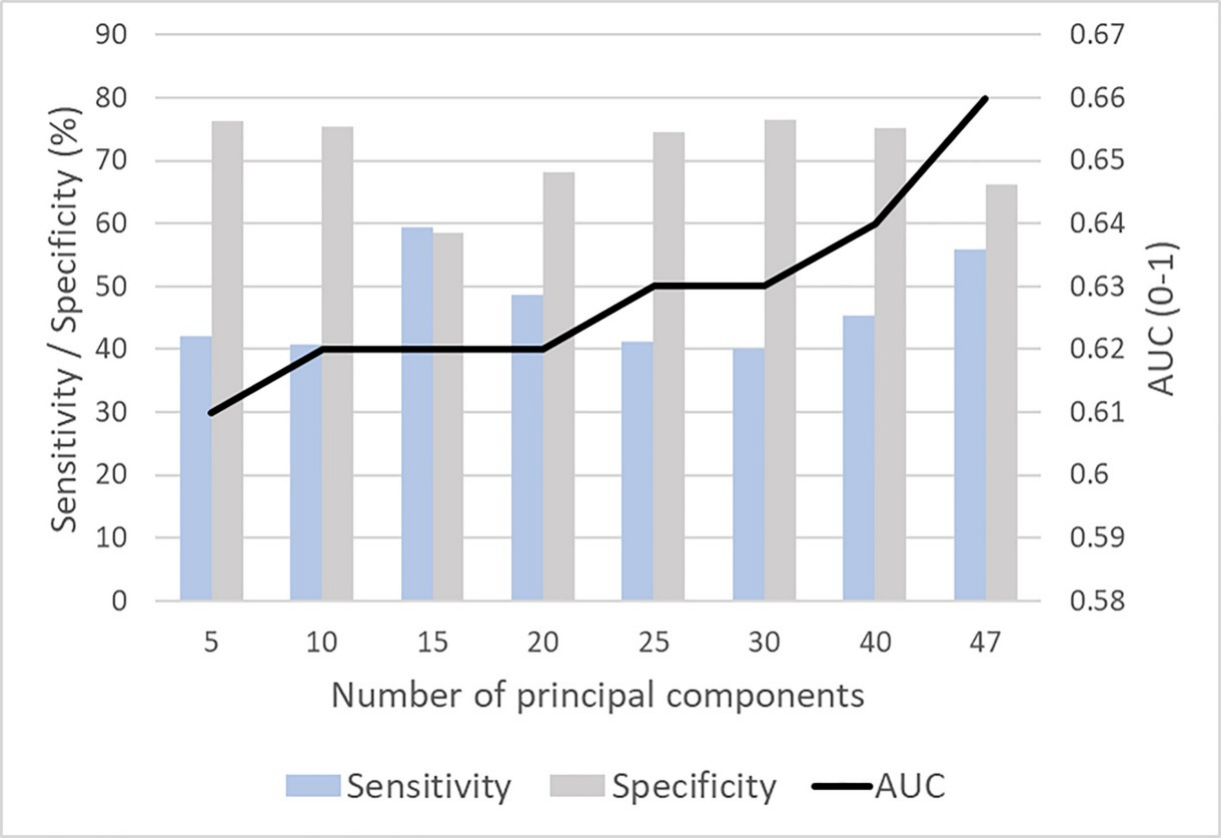
Performance of the multi-layer perceptron (MLP) prediction model on variables generated by principal component analysis.

The performance of the multi-layer perceptron (MLP) based prediction model using variables selected by different techniques is presented in Table 4. The initial prediction model with all of the available variables (*n* = 47) had an Area Under the receiver operating characteristic Curve (AUC) of 0.62 with sensitivity of 48.4% and specificity of 70.0%. In contrast, the statistical variable selection approaches produced an AUC in the range 0.58–0.60, with sensitivity ranging from 41.4% to 47.3% and specificity ranging from 62.5% to 71.4%. The prediction model based on the eight most relevant variables selected by the minimal Redundancy Maximum Relevance (mRMR) technique outperformed the other models with an AUC of 0.62, sensitivity 58.7% and specificity 60.6%. Fig 1 shows the performance of the MLP based prediction model using new variables (called principal components) generated by the Principal Component Analysis (PCA) algorithm. This resulted in further improvement in the AUC to 0.66 from 0.61 when the number of principal components increased from five to 47. We statistically compared the performance of the PCA based MLP model with the MLP model based on original 47 variables at 95% confidence level using paired t-test on 500 bootstrapped iterations. The PCA based model performed better with a mean difference of 0.00354 at a 95% confidence interval (0.0021–0.0050) with p<0.0001.

**Table 4:** Performance of the multi-layer perceptron prediction model for 30-day HF readmission or death in HF patients.

Variable selection approach	Number of variables	Sensitivity (%)	Specificity (%)	AUC
None	47	48.4	70.0	0.62
t-test	4	47.2	67.5	0.60
chi-squared test	8	47.3	62.5	0.58
t-test + chi-squared test	12	41.4	71.4	0.60
forward selection	8	23.2	85.3	0.56
backward selection	8	44.2	66.6	0.57
mRMR	8	58.7	60.6	0.62

HF = Heart Failure; MLP = Multi-Layer Perceptron; mRMR = minimal Redundancy Maximum Relevance.

## Discussion

In this study, we investigated different variable selection techniques to predict 30-day HF readmission or death in HF patients in the linked administrative health data. We report a matching performance by using only eight significant variables compared to the full set of 47 variables. In addition, we have also evaluated the use of a feature extraction ML technique called PCA, which generated new combinations of original variables/features that improved the performance of the model significantly.

The LACE score is a tool that was developed to predict 30-day unplanned readmissions from administrative data [20]. This score uses Length of hospital stay (L), Acuity of admission [A (emergency or not)], Comorbidity score (C), and the number of Emergency visits in the last 6 months (E) to predict the risk of readmission within 30 days of hospital discharge. The LACE score is not restricted to a particular disease and is used as a generic tool to predict unplanned readmissions for various health problems[21, 22]. However, when applied to HF patients, the LACE score was not able to predict 30-day readmissions (*p* = 0.199)[23]. Another tool to determine the risk of avoidable hospital readmissions is the HOSPITAL score [24]. This tool uses laboratory values such as hemoglobin and sodium level at discharge, and other patient attributes such as length of stay in hospital, number of hospital admissions in the previous year, and admission type to determine the risk of readmission. These clinical variables are not always integrated electronically in administrative health databases in many cases, thereby limiting the use of the HOSPITAL score to predict readmissions from routinely collected administrative data.

Several other ML models have been proposed to predict 30-day readmission or death for HF patients. Koulaouzidis et al. used the naive Bayes classifier on telemonitored data to predict HF readmissions [25]. They manually tested different combinations of a limited set of variables such as heart rate, systolic blood pressure, diastolic blood pressure, and body weight to develop their model. However, this manual approach only works with a small number of variables because the number of possible combinations grows exponentially when we have hundreds of input variables. Mortazavi et al. used more sophisticated ML algorithms such as random forest and support vector machines to predict 30-day and 180-day (all-cause and HF-specific) readmissions [26]. As the aim of their study was to compare the effectiveness of ML techniques against the traditional logistic regression, they used all of the available variables to develop their prediction model, without evaluating the discriminatory power of their input variables. Futoma et al. used a neural network to predict HF readmissions, given diagnosis and procedures of each hospital admission [27]. A portion of this work implemented a two-step variable selection process. The first step retained only those variables that passed a likelihood ratio test. The second step involved multivariate selection in a stepwise forward manner. They randomly ordered the variables that pass the likelihood ratio test in the first step. This random ordering is a limitation since it can potentially overlook a better ordering, thus harming the predictive performance. Our previous study showed the ability of machine learning techniques to predict HF readmission and death compared with standard statistical approaches which are used clinically[5]. Our current study extended this to explore the reduction and transformation of the original variables to determine the performance of the reduced and transformed feature sets to predict HF readmission and death.

The standard models to predict HF readmissions use variables, which are carefully selected by the clinical experts. In our study, we conceived a way to determine which variables have the most significant effect on predicting 30-day HF readmission or death from various selection techniques using statistical and ML domains. We were able to reduce the number of variables from the original 47 to 8 without compromising the predictive accuracy. The prediction model based on these 8 variables consisted of age, type of index admission, visit to an allied health professional in the last 6 months, length of hospital stay, use of antineoplastic and immunomodulating agents in the last 6 months, and history of HF, chronic kidney disease and depression. Its AUC of 0.62 was the same as the AUC of the model based on all 47 variables.

Variable extraction techniques empower us to determine if a combination of the original features can generate new features, which are more discriminating of the outcome than the original features. We used the PCA technique to transform the original variables into a new space based on the maximum variance in the data. This further significantly improved the AUC (from 0.61 to 0.66) when transformed variables are used for prediction. The addition of new variables improved the predictive performance because successive variables add to the variance of the data and this helps in separating the two outcome values. This performance was measured to be statistically significant (p<0.001) compared to the model that used the set of original variables.

## Conclusions

A ML model developed to predict 30-day HF readmission or death in HF patients using a reduced number of variables selected by feature selection techniques matched the performance of the model that used the full set of 47 variables. We also demonstrated that new variables generated by transforming the original variables, based on the variance in the data, further significantly improved the predictive ability of our ML model.

### Limitations

We limited our cohort to age 65 years and above to ensure that we captured all medication supplies for this patient group from the Pharmaceutical Benefits Scheme (PBS) dataset. However, this did not affect the capture of HF patients because the majority of HF admissions occur in patients aged 65 years and older, and they represent the more advanced forms of HF [28]. Our administrative data did not include specific clinical data such as laboratory values and blood pressure, which may have further improved the model performance. However, we have demonstrated the improvements that are possible when routinely collected administrative data are used to predict 30-day readmission and death.

## Supporting information

S1 Table Variables used in this study to predict 30-day readmission or death in the HF cohort. ARIA = Accessibility Remoteness Index of Australia; ATC = Anatomical Therapeutic Chemical index; BB = Beta Blockers; COPD = Chronic Obstructive Pulmonary Disease; GP = General Practitioner; HF = Heart Failure; PVD = Peripheral Vascular Disease; RAASi = Renin Angiotensin Aldosterone System index; SEIFA = Socio-Economic Indexes For Areas.
(PDF)
