# Supplementary material for: Feature selection and transformation by machine learning reduce variable numbers and improve prediction for heart failure readmission or death
Source: PLoS One. 2019 Jun 26;14(6):e0218760. doi: 10.1371/journal.pone.0218760 (PMC6594617; doi:10.1371/journal.pone.0218760)
Supplement: S1 Table — ARIA = Accessibility Remoteness Index of Australia; ATC = Anatomical Therapeutic Chemical index; BB = Beta Blockers; COPD = Chronic Obstructive Pulmonary Disease; GP = General Practitioner; HF = Heart Failure; PVD = Peripheral Vascular Disease; RAASi = Renin Angiotensin Aldosterone System index; SEIFA = Socio-Economic Indexes For Areas. (PDF) [file pone.0218760.s001.pdf]

S1 Table.

Feature Name	Type
**Patient demographics**	
Age	Continuous
Gender	Binary
Indigenous status	Binary
**Admission characteristics**	
Admission type (emergency, booked)	Binary
Length of stay (days)	Continuous
Time since last HF discharge	Continuous
**Past medical history (comorbidities)**	
HF admission in last 6 months	Continuous
Emergency department visit in last 6 months	Binary
Ischaemic heart disease	Binary
Hypertension	Binary
Atrial fibrillation	Binary
Diabetes	Binary
COPD	Binary
PVD	Binary
Stroke	Binary
Dementia	Binary
Depression	Binary
Cancer	Binary
Chronic kidney disease	Binary
Cardiogenic shock	Binary
Cardiomyopathy	Binary
Charlson comorbidity score	Categorical
**Socio-economics**	
SEIFA score	Categorical
ARIA score	Categorical
**Medication history**	
Drug group (BB, RASI)	Categorical
ATC codes	Binary
Out-of-hospital services (GP/Specialist/Allied health visit)	Binary

ARIA = Accessibility Remoteness Index of Australia; ATC = Anatomical Therapeutic Chemical index; BB = Beta Blockers; COPD = Chronic Obstructive Pulmonary Disease; GP = General Practitioner; HF = Heart Failure; PVD = Peripheral Vascular Disease; RASI = renin angiotensin system inhibitor; SEIFA = Socio-Economic Indexes for Areas.
